# Perioperative Anticoagulation and Risk Assessment: Building a Bridge Over a STREAM

**DOI:** 10.31729/jnma.8910

**Published:** 2025-03-31

**Authors:** Anil Harrison, Sushil Rayamajhi

**Affiliations:** 1 Department of Medicine, Midwestern University, Glendale, Arizona, USA; 2 University of Central Florida College of Medicine/HCA Florida West Hospital, Pensacola, Florida, USA

**Keywords:** *anticoagulation*, *bridging therapy*, *perioperative management*, *thromboembolism*, *risk stratification*

## Abstract

Perioperative care requires a comprehensive assessment of the risks of bleeding and thrombosis. The 2022 CHEST Guidelines offer an empirical foundation for customized treatment using clinical judgment and risk assessment based on CHA2DS2-VASC and HAS-BLED scores. Despite the ease of access to anticoagulation guidelines, decision-making remains challenging, particularly when determining the necessity of bridging therapy with unfractionated or low-molecular-weight heparin. To facilitate this decision, we provide a mnemonic, STREAM, that highlights high-risk situations that require bridging. These include recent stroke or transient ischemic attack, severe thrombophilia, rheumatic valvular disease, recent venous thromboembolism, atrial fibrillation with high CHA2DS2-VASC scores, and mechanical heart valves. This mnemonic offers physicians a methodical approach to optimize perioperative anticoagulation control while minimizing the risk of hemorrhage and thrombosis.

## INTRODUCTION

An essential component of perioperative management is the assessment of the patient's risk for thromboembolism, surgery/procedure-related bleeding, or the risk for a stroke/embolic phenomenon.^1,2^ The risk classification schemes in the 2022 CHEST Guidelines on Perioperative Antithrombotic Management aim to facilitate individualized patient care, but these guidelines are empiric and have not been prospectively validated.^3^

The guidelines are meant to be a starting point for risk assessment which also incorporates a clinical judgment pertaining to an individual patient's assessment for thromboembolism and bleeding. To bridge or not to bridge for a procedure with low-molecular-weight heparin or unfractionated heparin is justifiably determined by the thromboembolic risk versus the risk of bleeding, using the CHA2DS2-VASC score and the HAS-BLED.^4,5^

## To Bridge Across a Turbulent Stream

Anticoagulation is frequently recommended when the risk is high (>10% events/year). Although the guidelines for anticoagulation decisions can be accessed online, we developed a mnemonic to determine when a ‘bridge’ is essential. A bridge can be built over a bay, a river, or a STREAM). Below are the conditions, which folks can easily remember, under which you should consider bridging with unfractionated or low-molecular-weight heparin, due to the significant risks of thromboembolism. "To bridge across a turbulent STREAM:"

- (S) Stroke or TIA in the past 3-6 months
- (T) Thrombophilia (severe) example deficiency of protein C, protein S or Antithrombin 3, antiphospholipid antibodies, multiple abnormalities
- (A) Atrial fibrillation with CHA2DS2-VASC score of 5 or 6
- (R) Rheumatic valvular heart disease
- (E) Embolism/VTE in the last 3 months
- (M) Mechanical heart valve: any mitral valve prosthesis; any caged ball or tilting-disc aortic valve prosthesis

**Table 1:** Suggested Risk Stratification for Patient-specific Periprocedural Thromboembolism

Risk Category	Mechanical Heart Valve	Atrial Fibrillation (A)	Venous Thromboembolism
High	(M) Mitral valve with risk factors for strokeb	CHA2DS2VASc score ≥7 or CHADS2 score of 5 or 6	Recent (<3 mo and especially 1 mo) VTE (E)
(>10%/year risk of ATE	Caged ball or tilting disc valve in mitral/aortic position …		Severe thrombophilia (T) (deficiency of protein C, protein S or antithrombin, homozygous factor V Leiden or prothrombin gene G20210A mutation or double heterozygous for each mutation, multiple thrombophilia’s)
or >10%/month risk of VTE)	Mechanical valve (M)	Recent (<3 month) stroke or TIA	
	Recent (<3 month) stroke or TIA (S)	(R)Rheumatic valvular heart disease	Antiphospholipid antibodies Active cancer associated with high VTE riskc
Moderate	Mitral valve without risk factors for strokeb	CHA2DS2VASc score of 5 or 6 or	VTE within past 3-12 mo Recurrent VTE
(4%–10%/year risk of ATE or 4%–10%/month risk of VTE)	Bileaflet aortic valve with risk factors for strokeb		Non-severe thrombophilia (heterozygous factor V Leiden or prothrombin gene G20210A mutation) Active cancer or recent history of cancer
Low	Bileaflet aortic valve without risk factors for strokeb	CHA2DS2VASc score of 1-4 or	VTE >12 mo ago
(<4%/year risk of ATE or <2%/month risk of VTE)		CHADS2 score of 0–2 (and no prior stroke or TIA	

ATE, arterial thromboembolism; VTE, venous thromboembolism; TIA, transient ischemic attack; CHADS_2_=congestive heart failure, hypertension, age >75 years, diabetes mellitus, prior stroke or transient ischemic attack; CHA_2_DS_2_VASc = congestive heart failure, hypertension, age >75 years, diabetes mellitus, prior stroke or transient ischemic attack, vascular disease history, age >65 years, female sex. ^a^ Empiric risk stratification is a starting point for assessing perioperative thromboembolism risk; it should be combined with the clinical judgment that incorporates individual patient- and surgery/procedure-related factors. ^b^ Includes: AF, prior stroke/TIA during an anticoagulant interruption or other prior stroke/TIA, prior valve thrombosis, rheumatic heart disease, hypertension, diabetes, congestive heart failure, age ≥75 years. ^c^ Includes: pancreatic cancer, myeloproliferative disorders, primary brain cancer, gastric cancer, and esophageal cancer.
